# Role of Crystalloids in the Perioperative Setting: From Basics to Clinical Applications and Enhanced Recovery Protocols

**DOI:** 10.3390/jcm12185930

**Published:** 2023-09-12

**Authors:** Juan V. Lorente, Robert G. Hahn, José L. Jover, Enrique Del Cojo, Mónica Hervías, Ignacio Jiménez, Rafael Uña, Fernando Clau-Terré, Manuel I. Monge, Juan V. Llau, Maria J. Colomina, Javier Ripollés-Melchor

**Affiliations:** 1 Department of Anesthesiology and Critical Care, Juan Ramón Jiménez University Hospital, 21005 Huelva, Spain; 2 Fluid Therapy and Haemodynamics Working Group of the Haemostasis, Fluid Therapy and Transfusional Medicine of the Spanish Society of Anesthesiology and Resuscitation (SEDAR), 28003 Madrid, Spain; 3 Karolinska Institute, Danderyds Hospital (KIDS), 171 77 Stockholm, Sweden; 4 Department of Anesthesiology and Critical Care, Verge del Lliris Hospital, 03802 Alcoy, Spain; 5 Department of Anesthesiology and Critical Care, Don Benito-Villanueva de la Serena Health District, 06400 Don Benito, Spain; 6 Department of Anesthesiology and Critical Care, Gregorio Marañón General University Hospital, 28007 Madrid, Spain; 7 Paediatric Anaesthesiology Section, Spanish Society of Anesthesiology and Resuscitation (SEDAR), 28003 Madrid, Spain; 8 Department of Anesthesiology and Critical Care, Virgen del Rocío University Hospital, 41013 Seville, Spain; 9 Department of Anesthesiology and Critical Care, La Paz University General Hospital, 28046 Madrid, Spain; 10 Vall d’Hebron Institut Recerca, Vall d’Hebrón University Hospital, 08035 Barcelona, Spain; 11 Department of Anesthesiology and Critical Care, Doctor Peset Hospital, 46017 Valencia, Spain; 12 Department of Anesthesiology and Critical Care, Bellvitge University Hospital, University of Barcelona, 08907 Barcelona, Spain; 13 Department of Anesthesiology and Critical Care, Infanta Leonor Hospital, 28031 Madrid, Spain; 14 Department of Toxicology, Universidad Complutense de Madrid, 28040 Madrid, Spain

**Keywords:** fluid therapy, postoperative complications, enhanced recovery after surgery

## Abstract

Perioperative fluid management, a critical aspect of major surgeries, is characterized by pronounced stress responses, altered capillary permeability, and significant fluid shifts. Recognized as a cornerstone of enhanced recovery protocols, effective perioperative fluid management is crucial for optimizing patient recovery and preventing postoperative complications, especially in high-risk patients. The scientific literature has extensively investigated various fluid infusion regimens, but recent publications indicate that not only the volume but also the type of fluid infused significantly influences surgical outcomes. Adequate fluid therapy prescription requires a thorough understanding of the physiological and biochemical principles that govern the body’s internal environment and the potential perioperative alterations that may arise. Recently published clinical trials have questioned the safety of synthetic colloids, widely used in the surgical field. A new clinical scenario has arisen in which crystalloids could play a pivotal role in perioperative fluid therapy. This review aims to offer evidence-based clinical principles for prescribing fluid therapy tailored to the patient’s physiology during the perioperative period. The approach combines these principles with current recommendations for enhanced recovery programs for surgical patients, grounded in physiological and biochemical principles.

## 1. Introduction

Intravenous fluid infusion is a ubiquitous therapy in perioperative patient management [1]. The misguided management of perioperative fluid therapy may result in postoperative complications, especially in high-risk surgical patients [2]. Such postoperative complications may result in an increase in surgical procedure-associated mortality [3]. Therefore, individualized perioperative fluid therapy regimens should be planned on the basis of the patient’s characteristics and clinical status and the type of surgery to be performed [4].

Crystalloid fluids have traditionally been used to prevent perioperative hypovolemia. Not until this millennium was it realized that fluid overload is a common problem and that the “therapeutic window” for crystalloid fluids is actually quite narrow. In fact, fluid therapy protocols must carefully consider the optimal amount of fluid to be infused in different clinical situations [5]. Despite the extensive literature on this subject, significant variability persists regarding the optimal fluid volumes to be administered. Fluid overload causes interstitial edema, which has been associated with postoperative complications in the lungs, cardiovascular system, gastrointestinal (GI) tract, and kidneys [6,7]. The infusion of >2 L of Ringer’s solution results in longer gastrointestinal recovery times [8]. If 0.9% saline is chosen, pH will be at the lower end of the normal range [9,10,11]. The infusion of 3 L is associated with more complications, particularly with GI surgery [12]. The infusion of >6 L may cause fatal pulmonary edema [13], and 9–10 L of fluid has been reported to break up interstitial tissues with pitting edema, lacunae of fluid in critical organs such as the heart, and life-threatening functional disturbances [14,15]. Consequently, numerous clinicians have embraced perioperative fluid restriction as standard practice [7]. However, the RELIEF trial (restrictive versus liberal fluid therapy in major abdominal surgery) that compared restrictive versus liberal fluid therapy in major abdominal surgery showed that a restrictive fluid regimen was associated with more abundant postoperative elevations of plasma creatinine consistent with acute kidney injury (AKI) [16]. Regardless of whether these elevations were transient or not, this finding has prompted the abandonment of the restrictive approach during major surgery.

**Figure 1:**
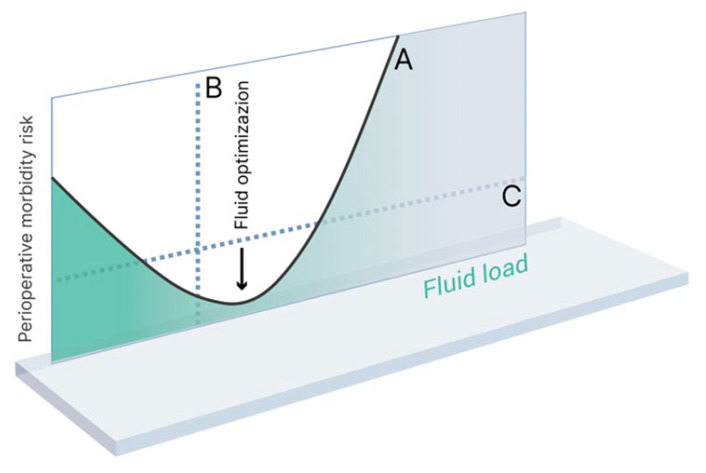
Fluid load versus perioperative morbidity risk (modified from Bellamy) [18]. Curve A represents the hypothesized line of risk. Broken line B represents a division between patient groups in a ‘wet vs dry’ study. Broken line C represents a division between patient and groups in an ‘optimized vs non-optimized’ study.

Therefore, one of the primary objectives of perioperative fluid therapy is to maintain adequate circulating blood volume while preventing both hypervolemia and hypovolemia, and AKI, as these conditions are all known to contribute to unfavorable postoperative outcomes [17]. See Figure 1.

Another pivotal function of perioperative fluid therapy is to preserve the homeostasis of plasma and prevent disruptions to the internal environment that could adversely impact vital organs [19]. Infused fluids have been linked to metabolic alterations, which have, in turn, been associated with the occurrence of postoperative complications [20]. To ensure the preservation of the homeostasis of plasma, a perioperative fluid therapy regimen must allow for the careful selection of the most appropriate fluid type.

The aim of this article is to provide an overview of the history, basic physiology, and currently recommended use of crystalloid fluids during the perioperative period.

## 2. Historical Evolution of Crystalloid Fluids

The infusion of electrolyte-based intravenous fluids has become a standard practice in healthcare settings. The origins of modern fluid therapy can be traced back to the cholera pandemic that brought havoc to India in 1831. As a result of trade movements, the disease quickly spread to Europe, including the British Isles, resulting in over 23,000 individuals dying in Britain from an undiagnosed illness [21].

During that time, a young Irish physician named William Brooke O’Shaughnessy journeyed to England to aid in combating the pandemic. He described the disease caused by the Vibrio Cholerae bacterium as “sudden, deadly, overwhelming, the living death”. The existing remedies at the time, such as bloodletting, leech therapy, and powerful cathartics, had proven ineffective [22]. However, O’Shaughnessy, drawing on his knowledge of chemistry, observed that large quantities of water, sodium, chloride, and bicarbonate disappeared from patients’ blood and showed up in their feces. He published his findings in The Lancet, emphasizing the importance of promptly replenishing the water and salt lost by patients. Following O’Shaughnessy’s discoveries, cholera patients were subsequently treated with intravenous saline fluids [23].

Two months after the publication of O’Shaughnessy’s paper, another British doctor named Thomas Latta, inspired by O’Shaughnessy’s work, performed the first intravenous fluid resuscitation in May 1832. Latta used a hypotonic aqueous mixture of sodium, chloride, and bicarbonate, saving the lives of 8 of the 25 treated patients [24].

Intravenous therapy has been the subject of controversy since its inception. Some clinicians hailed it as a “miraculous and supernatural agent”, while others in the medical profession condemned the idea of violating the sanctity of the human body. The latter view seems to have prevailed, as O’Shaughnessy’s rehydration therapy was ignored during the six cholera pandemics of the 19th century. Of course, the lack of safe materials capable of ensuring infection-free intravenous infusions was also a likely culprit [25].

Over the next 80 years, multiple scientific breakthroughs were made, largely driven by an improved understanding of cardiovascular pathophysiology. Around 1876, Sydney Ringer developed a physiological calcium-rich solution for intravenous perfusion based on his discovery that the infusion of such calcium-rich fluids into the hearts of experimental frogs enhanced their contractility. These ground-breaking findings fueled the advancement of intravenous infusion therapy [26]. In 1934, Alexis Hartman added sodium lactate to Ringer’s solution to address the high levels of acidosis observed in treated patients, giving rise to the Ringer lactate solution that is still in use today [27].

The first mention of a solution similar to 0.9% saline dates back to an article by WS Lazarus-Barlow published in 1896. Based on experiments by Dr. Hamburger, Lazarus-Barlow found that a saline concentration of 0.92% was normal in mammals. Hamburger had demonstrated the isotonicity of 0.9% saline with human plasma in a series of in vitro studies, leading to the misconception that 0.9% saline was physiologically saline [19].

In recent years, authors have drawn on past experience and on current evidence to search for intravenously infused fluids that closely resemble the electrolytic composition of plasma and can keep patients’ physiological parameters and internal environment as close as possible to normal levels. In this regard, balanced solutions have garnered significant interest and have largely replaced 0.9% saline as the standard of care in the operating room [28]. However, 0.9% seems still to be the most widely used crystalloid fluid in European hospitals [29,30]

## 3. The Biochemical Basis of Fluid Use in the Surgical Setting

### 3.1. Nomenclature and Basic Biochemical Concepts for Fluid Prescription

A solution is the outcome of a homogeneous mixture of multiple substances, with the solvent, typically water in biological contexts, being the most abundant component. Substances dissolved in water are referred to as solutes. Water can form solutions with various solutes [31]. The quantitative relationship between the solute and solvent in a solution, known as concentration, can be expressed in different ways. However, none of these expressions has been officially adopted by the International System of Units (SI) [32].

The osmotic effect of a solution is determined by the number of dissolved particles it contains, regardless of their weight, electrical charge, or valency, as one mole of any element consists of the same number of molecules (the Avogadro constant) [33]. One osmole represents the number of particles in a one-gram molecular weight of an undissociated solute. The concentration of a solution can be defined as the amount of solute present in a given quantity of solution or solvent. Osmolarity, expressed as mOsm/l, indicates the number of osmoles of a specific solute per liter of solution and reflects the concentration of particles in a given solution [34]. On the other hand, osmolality represents the number of osmoles per unit of solvent, signifying the concentration of osmotically active particles per unit of mass. Lastly, the tonicity of a solution refers to the effective osmolarity, which determines its behavior within a living organism. More specifically, tonicity predicts the impact of a solution on the volume of an osmotically balanced cell and relies on the relative concentration of solutes that are impermeable to the cell within the solution [35]. Sodium is the most critical solute for determining the tonicity of a solution [36].

Consequently, it is possible for a hyperosmotic solution to be hypotonic. One example is ethanol, which greatly increases osmolality but easily enters cells and, therefore, does not redistribute water. Many amino acids, such as glycine, enter cells with only minor difficulty. Another example is a 20% glucose solution, which, despite being hyperosmotic due to its glucose content, acts as “free water” in vivo after the glucose has been metabolized. 

### 3.2. Electrolytic and Acid–Base Balance and Crystalloid Infusion 

Acute changes in blood pH induce regulatory effects on protein and enzyme structure and function, leading to changes in cell function such as glycolysis, gluconeogenesis, mitosis, and DNA synthesis, among others. Hence, it is important to understand the concurrence of elements governing pH maintenance within physiologic boundaries, such as HCO3^−^, H^+^, phosphates, albumin, Na^+^, K^+^, and others, as they enable the preservation of complex and efficient cell functions related to the acid–base balance [37].

Two chemical models have been developed to elucidate the ionic behavior of the acid–base balance:

1. The traditional model, known as the Henderson-Hasselbach equation [38];
2. Stewart’s theory, based on the Law of Conservation of Matter, the Law of Mass Action (stating that any incompletely dissociated substance reaches a state of dissociation equilibrium), and the Principle of Electroneutrality [39].

**Figure 2:**
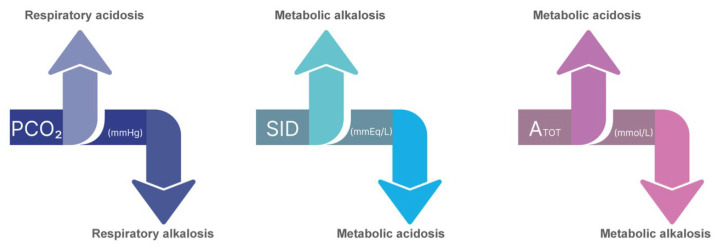
Variables that independently influence the pH of biological fluids according to Stewart’s theory. A_TOT_—total weak acid concentration; SID—strong ion difference; P_a_CO_2_—partial CO_2_ pressure. If the SID of the infused solution is below 24 mEq/L, it will result in metabolic acidosis, and if it is above 24 mEq/L, it will induce metabolic alkalosis. An increase in A_TOT_ is linked to acidosis, while a decrease in A_TOT_ leads to alkalosis.

In the traditional approach using the Henderson-Hasselbalch equation, bicarbonate independently determines pH. In Stewart’s approach, however, bicarbonate is only one of several dependent ions and is thus conceived as a marker, not a mechanism. According to the latter model, three independent variables can govern the acid–base behavior of bodily fluids if they exert control over the physical medium of these fluids. These variables are the total weak acid concentration (ATOT), the strong ion difference (SID), and the partial CO_2_ pressure (PaCO_2_) [39]. See Figure 2. 

As the influence of fluid therapy on acid–base balance involves the metabolic component, the respiratory component is beyond the scope of this review.

The approximate amount of ATOT is calculated using the following equation [40]:

2.5 × Albumin (g/dL) + 0.6 × Phosphate (mg/dL)

An increase in ATOT is linked to acidosis, while a decrease in ATOT leads to alkalosis. A decrease in ATOT is caused by hypoalbuminemia. The calculation formula for ATOT emphasizes the significant impact of albumin. In critically ill patients, hypoalbuminemia is primarily caused by fluid overload, endothelial leak, and nutritional deficiencies [41]. While it may appear that alkalosis resulting from hypoalbuminemia could prevent the development of acidosis, this notion is inaccurate [42]. As a compensatory mechanism, the decrease in ATOT and the increase in pH prompt the retention of chloride, which subsequently decreases the SID. Consequently, a low SID indicates reduced tolerance to acid load [43]. The contribution of inorganic phosphate is relatively minor and often negligible, except in cases of hyperphosphatemic acidosis due to renal failure [44].

Certain elements, namely Na^+^, K^+^, Ca^2+^, Mg^2+^, and Cl^−^, exclusively exist in fully ionized form within body fluids. At physiological pH, this phenomenon primarily applies to anions with pKa values ≤4, especially in quantitative terms. Examples of such anions include sulfate, lactate, and beta-hydroxybutyrate. Stewart referred to compounds that display this characteristic as strong ions, which originate from substances that completely dissociate in water [45]. The main strong ions present in blood include Na^+^, K^+^, Ca^2+^, Mg^2+^, and Cl^−^. Notably, body fluids exhibit an abundance of strong cations, quantified by Stewart as the SID. In simple terms, this so-called apparent SID (SIDa), where the contribution of weak acids is not considered, can be calculated through subtracting the concentration of strong anions from that of strong cations [46], and it is typically expressed in mEq/L. Lactate is also considered a strong ion, although only at physiologic pH.

For normal plasma, SIDa can be calculated using the following formula:

SIDa = ([Na^+^] + [K^+^] + [Mg^2+^] + [Ca^2+^]) − ([SO4^2−^] + [(Cl^−^) + (Lactate^−^)] (1)

The in vitro SID of all fluid (SID in a bag) is zero according to the law of electroneutrality. However, when balanced crystalloids are mixed with plasma, the buffers are eliminated. Due to this gap, the in vivo SID becomes higher than zero. It can be hypothesized that administering a crystalloid with a similar in vivo SID to that of normal plasma (i.e., 42 mEq/L) will not alter the plasma SID. However, when crystalloid solutions are infused, they typically lead to the dilution of weak acids, especially albumin, which may increase the patient’s susceptibility to metabolic alkalosis [47]. Considering this, there is evidence suggesting maintenance of plasma SID can be achieved when the infused crystalloid has a SID similar to that of normal bicarbonate (24 mmol/L) or to the patient’s bicarbonate concentration. If the SID of the infused solution is below 24 mEq/L, it will result in metabolic acidosis, and if it is above 24 mEq/L, it will induce metabolic alkalosis [48]. 

However, for the determination of the global metabolic component of the acid–base equilibrium using the Stewart model, the strong ion gap (SIG) must calculated, and for this, the apparent strong ion difference apparent (SIDa) and the effective strong ion difference (SIDe) must be known first [49]. 

SIDe is determined by the formula:

SIDe = (HCO^3−^) + (Albumin) + (Phosphate) (2)

in which weak acids are also included. Therefore, to calculate SIG, the difference between SIDa and SIDe is determined (SIG = SIDa − SIDe) and indicates the presence of other non-measured weak anions such as keto acids, sulphates, urates, citrate, pyruvate, acetate, and gluconate. Accordingly, hyperalbuminemia results in a reduction in the SIG, causing acidosis, whereas hypoalbuminemia causes metabolic alkalosis because it increases SIG [41]. No standard range for SIG has been mentioned in previous evidence. It is expected to be below 2 mEq/L, but in most studies, it was determined to be approximately 5 mEq/L for critical patients [50]. 

### 3.3. Crystalloids: Definition and Classification

Crystalloids are aqueous solutions that consist of inorganic cations and organic and inorganic anions (Table 1). Saline, for instance, contains only Na^+^ and Cl^−^ ions at supraphysiological levels (154 mmol/L). Its osmolarity and osmolality closely resemble those of plasma [51].

Balanced crystalloids are solutions designed to minimize disruptions in the acid–base balance caused by fluid infusion. In these solutions, chloride anions are replaced by sodium bicarbonate or other buffers. Initially, balanced crystalloids achieved this through replacing chloride with bicarbonate anions. However, due to the instability of bicarbonate-containing solutions in gas-permeable bags, modern balanced crystalloids now incorporate buffers, which are often rapidly metabolized or excreted [27]. The metabolism of buffers varies depending on their location, oxygen consumption, and their ability to generate CO_2_. The most commonly used buffers in crystalloids include the following:

(a) L-Lactate is metabolized primarily in the liver within approximately one hour. However, one major disadvantage of lactate becomes particularly obvious in cases of peripheral tissue hypoxia, as the accumulation of l-lactate can cause significant interference, making it difficult to accurately measure systemic perfusion. It is not recommended for use in patients with diabetes [52,53]
(b) Acetate is typically metabolized within 15 min, al.though the exact duration may vary among patients. After entering the bloodstream, it undergoes swift absorption by cells and is subsequently metabolized into acetyl-coenzyme within those cells. This metabolic transformation is possible within 15 min in all body cells that engage in aerobic oxygen metabolism, although the exact duration may vary among patients. The most elevated metabolic rates are observed in the heart, liver, skeletal muscles, and kidneys. It does not pose a risk of buildup. Acetate is associated with the lowest oxygen consumption and the highest carbon dioxide production. In large quantities, such as in dialysis applications, it may lead to vasodilation and reduced cardiac contractility. However, the amount of acetate in balanced fluids is generally low, mitigating these concerns [52,54].
(c) Gluconate undergoes partial but rapid metabolism in the liver, with around 80% being cleared through the kidneys. It is associated with the highest metabolic consumption of oxygen and the highest production of carbon dioxide [55]. Gluconate is widely used in the food industry to counteract sour taste (juices, wine, and bakery products). Gluconate has a protective effect against dysfunctions and oxidative injuries following cardiac ischemia. However, it is worth noting that some authors have reported a theoretical risk of gluconate contributing to the development of bradycardia, although this risk has not been encountered in clinical practice [56]. Gluconate only marginally changes the bicarbonate concentration in plasma. The role of gluconate in PlasmaLyte is to maintain electroneutrality despite its lower chloride content (98 mmol/L versus 110 mmol/L in Ringer’s) [57].
(d) Malate: While malate is metabolized at the muscular level [56], there is limited available literature on this subject.

### 3.4. The Patient’s Internal Environment during the Perioperative Period: Importance of Sodium and Chloride

Surgery induces comprehensive lifestyle changes, demanding higher performance levels from patients, not only due to social or personal reasons but also because of underlying conditions and the physiological stress caused by anesthesia and surgery themselves [58]. In response to this heightened demand, the human body activates a coordinated set of systems (neuroendocrine, metabolic, and immunologic) to facilitate the adaptive changes needed to restore homeostasis and repair potential injuries. Stimuli such as pain or hypoxia, likely to occur during the perioperative period, can perpetuate the surgical stress response [59].

Neuroendocrine changes resulting from stress manifest through a dual effector system: the hypothalamic–pituitary–adrenal (HPA) axis and the sympathetic–adrenal–medullary (SAM) axis. The alterations caused by the HPA axis are the ones exerting the strongest impact on the internal environment. The HPA axis triggers the secretion of vasopressin (ADH) in synergy with the corticotropin-releasing hormone, which acts on the pituitary gland and promotes the release of corticotropin (ACTH) into systemic circulation. ACTH stimulates the production and release of cortisol and aldosterone, affecting various physiological processes [60]. Additionally, surgical stress has been linked to an increased release of renin [61].

Due to the aforementioned hormonal changes, surgery leads to water and salt retention in the body. Moreover, the anesthesia-associated reduction in the arterial pressure unloads the baroreceptors, which increases the sympathetic nerve activity in the kidneys and also results in strong water and sodium retention [62,63]. Compared to the conscious state, the diuretic response to a load of crystalloid solution during anesthesia is inhibited by 80–90% [64]. The anesthesia-induced restriction of the diuretic response to volume loading ceases within one hour after awakening from general anesthesia [65].

Sodium and chloride are two essential ions that play a crucial role in maintaining the internal environment. Sodium is a major determinant of plasma tonicity and is consequently essential for proper osmoregulation. Despite this, the human kidney lacks an active sodium excretion mechanism [66]. The hormonal and internal environment changes caused by this saline infusion typically persist for up to nine days [67]. Moreover, restoring the internal environment after excessive sodium intake is not a benign process. On the contrary, it requires energy expenditure because urea needs to accumulate in the renal medullary interstitium to generate sufficient osmotic pressure for the production of more concentrated urine [68]. These internal environment alterations also have notable clinical effects. For instance, a study by Lobo et al. [69] randomized patients undergoing colon resection surgery into two groups: one receiving standard postoperative maintenance fluid therapy with a sodium intake of at least 154 mmol per day and the other restricted to 77 mmol per day. The group with positive salt-water balance experienced delayed recovery of gastrointestinal function and significantly longer hospital stays [69]. The clinical consequences of internal environment alterations extend beyond the kidneys. A prospective observational study involving 50 mechanically ventilated patients demonstrated that a positive sodium balance was associated with reduced PaO_2_/FiO_2_ at 24 h and prolonged assisted ventilation [70]. Furthermore, some authors have found a correlation between increased natremia during the first 48 h in the intensive care unit (ICU) and higher in-hospital mortality rates, except in patients presenting with hyponatremia upon admission [71].

The optimal functioning of organs, particularly the kidneys, relies on maintaining appropriate chloride plasma concentrations. Although there is no clear limit for diagnosing hyperchloremia, the most commonly accepted is Cl > 110 mEq/L. Hyperchloremia caused by underlying diseases or fluid therapy is common in ICUs, within 25–45%. Hyperchloremia often occurs together with acidosis. In this case, the pH is lowered by decreased SID and not only by hyperchloremia. Hyperchloremia can also appear with alkalosis; this situation occurs in the presence of greater hypernatremia and therefore with increased SID. This fact highlights the presumably greater importance of relative chloremia compared to absolute chloremia, although it is a controversial issue [72]. These changes in the internal environment have clinical consequences and do not necessarily require the infusion of large amounts of fluids. The administration of two liters of saline solution through infusion has been linked to the development of hyperchloremic metabolic acidosis [9,10,11,73]. This condition is accompanied by a reduction in the flow rate within the renal artery, resulting in decreased renal cortical perfusion. Furthermore, it has been observed that excessive chloride levels exceeding 115 mmol/l raise renal vascular resistance by 35%, consequently leading to a 20% decline in the glomerular filtration rate. Consequently, patients receiving chloride-rich fluid therapy are at a higher risk of renal failure compared with those receiving low-chloride fluid therapy [20]. Additionally, this high-chloride approach has also demonstrated the potential to cause splanchnic hypoperfusion [74].

## 4. Pathophysiology of Fluid Therapy

**Figure 3:**
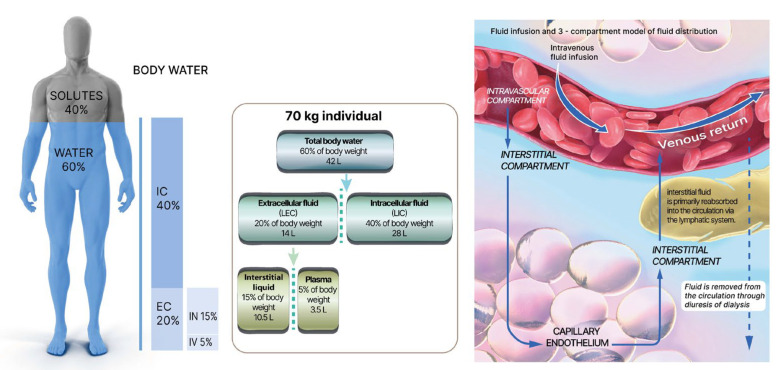
Fluid infusion and distribution of body fluid in the 3 compartments (modified from Zampieri et al.) [76].

In normal conditions, approximately 60–65% of an adult’s body weight consists of water, with this percentage gradually decreasing during childhood until the age of ten, when it becomes comparable to that of adults. Neonates, on the other hand, may have water levels as high as 75–80%. In women, the percentage of water is slightly lower, around 55%. Water is dynamically distributed among three bodily compartments: two-thirds are allocated to the intracellular compartment, while the remaining third is distributed to the extracellular space, which can be further divided into the interstitial space (75–80%), the intravascular space (5–6%), and the transcellular space, housing specific types of fluid, such as pleural or pericardial fluid, crucial for organ integrity [75]. See Figure 3.

### 4.1. Fluid Shifts across the Cell Membrane

The intracellular compartment is separated from the extracellular fluid by a semipermeable cell membrane. Fluid shifts between these compartments occur by virtue of osmosis, simple diffusion, and transmembrane channels. Changes in tonicity within either compartment allow water to flow freely between them. Regarding simple diffusion, the cell membrane permits the passage of a few solutes, which can move effortlessly down a concentration gradient [77]. On the other hand, the diffusion of electrolytes is an energy-dependent process facilitated by transmembrane channels [78].

### 4.2. Diffusion across Capillary Walls

The movement of solutes or water across capillary walls predominantly occurs through filtration and simple diffusion. Volume kinetic studies suggest that the passage of crystalloid fluid across the capillary wall occur 4–5 times faster than the urine flow in the conscious state [65,79], and the difference is greater during general anesthesia [64,80]. The classical understanding of fluid shifts between the interstitium and intravascular space was based on the Starling principle. According to this principle, differences in hydrostatic and oncotic pressures on either side of the endothelium drive plasma extravasation from the arterial side to the interstitium through intercellular pores in the microcirculation. The majority of this plasma is subsequently reabsorbed toward the inner part of the vessel on the venous side, while a small portion returns to circulation via the lymphatic system [81]. However, advances in our understanding of the glycocalyx structure and functions as well as fluid resorption at the venular side have led to a revised version of the Starling principle [82].

The glycocalyx, a dense network of glycoproteins and associated proteoglycans attached to the luminal side of endothelial cells, forms a primary barrier preventing macromolecules from entering the sub-glycocalyx space immediately below, which is mostly devoid of proteins. The forces governing plasma exchange between the vascular system and the interstitium are the differences in hydrostatic and osmotic pressures between the interstitial space and the subglycocalyx region, covering the intercellular pores [83]. Moreover, the net fluid flux at the venular side of capillaries is considerably lower than originally proposed by the Starling principle. Factors such as inflammation, ischemic-reperfusion phenomena, hypervolemia, sepsis, trauma, or perioperative factors can contribute to glycocalyx degradation [84]. The movement of fluids from the intravascular space to the interstitium can occur under two different circumstances. First, fluids that are virtually protein-free can reach the interstitium even when the vascular barrier remains intact. Second, when the integrity of the glycocalyx is compromised, protein-containing fluids can access the interstitial space at a higher rate than normal. This emphasizes the crucial importance of preserving glycocalyx integrity in any perioperative fluid therapy protocol [85].

### 4.3. The Lymphatic System

The lymphatic system plays a vital role in the regulation of fluid balance, immune defense, and overall health. It is composed of lymphatic vessels, lymph nodes, lymphoid organs, and lymphoid tissues, forming a transportation network for lymph. Lymph is a clear fluid that carries nutrients, waste products, and immune cells. Lymphatic vessels run parallel to blood vessels throughout the body, ranging in size from microscopic capillaries to larger collecting ducts [86]. The lymphatic capillaries present in most tissues and organs have unique structural features that allow them to collect interstitial fluids, proteins, and cellular debris. These capillaries consist of thin endothelial cells with overlapping edges, creating one-way mini-valves that enable fluid entry while preventing backflow. Additionally, the lymphatic vessels are equipped with smooth muscle cells that contract rhythmically, propelling the lymph forward [87].

The primary function of the lymphatic system is to maintain fluid balance in the body. Excess fluid, proteins, and cellular waste products that escape from the blood capillaries into the interstitial space are collected by the lymphatic capillaries. This fluid, now known as lymph, is transported through the lymphatic vessels, filtered in lymph nodes, and eventually returned to the bloodstream. Through preventing the accumulation of excess fluid, the lymphatic system promotes tissue health and prevents swelling, also known as edema [86].

Normal lymphatic pump function is determined by the intrinsic properties of lymphatic muscle and the regulation of pumping by lymphatic preload, afterload, spontaneous contraction rate, contractility, and neural influences. Lymphatic contractile dysfunction, barrier dysfunction, and valve defects are common themes among pathologies that directly or indirectly involve the lymphatic system [88].

The movement of interstitial fluid into the lymphatic system is facilitated by two types of tissue forces: extrinsic and intrinsic forces [89]. Extrinsic forces are generated by the movement of tissues, including skeletal muscle contractions, breathing, peristalsis, arterial pulsations, and external massage. These actions increase tissue pressure, leading to the opening of primary lymphatic vessels. These vessels, similar to capillaries, have walls composed of loosely connected endothelial cells. When tissue pressure rises, these cells open, allowing fluid to enter the lymphatic capillaries. Intrinsic forces, on the other hand, refer to the contractility of smooth muscle cells in the lymphatic system. Lymphatic smooth muscle cells exhibit characteristics of both smooth muscle and cardiac muscle cells. They contract spontaneously and exert contractile forces that are influenced by factors such as preload, afterload, and contractility with unidirectional valves; the intrinsic pumping of lymphatic smooth muscle cells determines the rate of lymph flow [90]. Laboratory studies demonstrate that anesthetic agents inhibit lymphatic pumping [91], which creates a modest maldistribution of fluid in perioperative patients. The clinically apparent maldistribution of fluid may develop in shock states and inflammatory conditions. Volume kinetic studies have demonstrated a significant reduction in the return flow of fluid from the interstitial space to the plasma, likely through the lymphatics [92]. Conditions such as transurethral resection syndrome, sepsis and pre-eclampsia are characterized by hypovolemia, hypoalbuminemia, and peripheral edema. Inflammatory molecules have been observed to contribute to the improper distribution of fluid during acute inflammation, such as sepsis and burn, through decreasing interstitial hydrostatic pressure [93,94]. Impaired lymphatic pumping exacerbates interstitial edema and hypovolemia. During sepsis and sustained inflammation, increased levels of nitric oxide inhibit the pumping of lymphatic smooth muscle cells, contributing to lymphatic failure [95,96,97]. The delayed return of albumin-rich lymph likely causes the misallocation of albumin during inflammatory conditions, as the production rate of albumin remains normal during major abdominal surgery and sepsis. The combined effects of low interstitial fluid pressure and impaired lymphatic pumping lead to a significant expansion of the interstitial space [92,98,99].

### 4.4. Kinetics of Crystalloid Fluid

**Figure 4:**
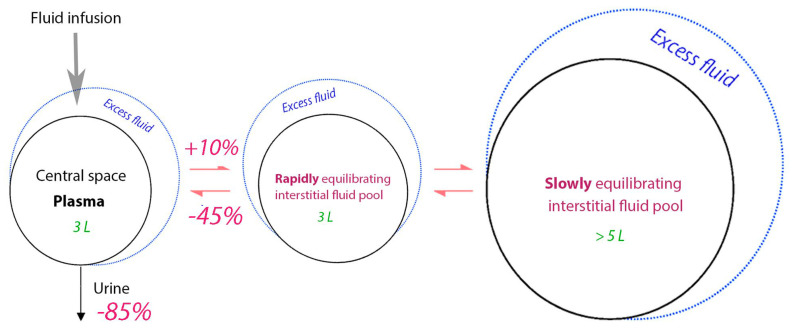
Changes in the distribution rates of crystalloid fluid resulting from general anesthesia as compared to the conscious state.

Volume kinetic analysis of crystalloid fluid suggests that the infused volume distributes between three body fluid compartments: a central volume (the plasma) and one fast-exchange and one slow-exchange interstitial compartment. An infusion of <500 mL distributes only in the central space while larger volumes equilibrate between the plasma and the two interstitial spaces. The entrance of fluid to the slow-exchange space is slow and creates a typical biphasic plasma volume (PV) expansion curve which gives crystalloid fluid a fairly effective PV expansion during infusion (approximately 50% of the infused volume). The distribution phase lasts only for 25–30 min, which means that the strong PV expansion is of short duration [100,101]. However, approximately 20% of the infused volume remains in the intravascular space for a long time if there is effective lymphatic flow. Only small amounts of Ringer´s solution enter the intracellular space [100,102]. See Figure 4.

The data were derived from a volume kinetic analysis of 217 infusion experiments where 1.5 L (mean) of Ringer´s solution was infused over 30 min in 106 non-dehydrated volunteers and 111 patients undergoing surgery. The slowly equilibrating interstitial fluid pool accumulates more volume during general anesthesia due to the impairment of the return flow to the plasma, which creates a “lock-out” effect. Preliminary data suggest that the remote pool shifts volume to the plasma during blood loss (unpublished).

## 5. Fluid Therapy Indications in the Surgical Setting

Fluids prescribed for therapy should be considered medications in their own right. Therefore, fluid therapy should be based on the careful selection of a specific product administered at the appropriate dose, tailored to address a specific indication over a defined period. As with any medication, it is crucial to identify any adverse events associated with fluid therapy [103].

When prescribing fluids, it is essential to consider factors such as electrolytic composition, the route of administration, osmolality, tonicity, and the presence of buffers. Additionally, the patient’s clinical status, the potential for renal and/or liver failure, acid–base balance, capillary leaks, water balance, albumin levels, and other relevant parameters should be evaluated to guide a physiologically appropriate prescription [104].

**Figure 5:**
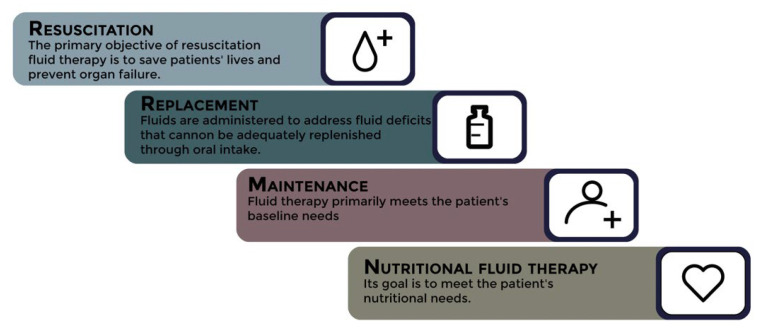
Fluid therapy indications in the surgical setting.

Furthermore, fluid selection varies depending on the purpose of the therapy, which includes four indications for fluid administration: resuscitation, replacement, nutrition, or maintenance, either individually or in combination [105]. See Figure 5.

The primary objective of resuscitation fluid therapy is to save patients’ lives and prevent organ failure [6]. It addresses both absolute and relative hypovolemia, which can manifest as either normal hypoperfusion markers or a true state of shock [106].

In certain situations, optimizing preload can be beneficial in achieving an optimal volumetric state and striking a balance between oxygen supply and demand. Ensuring the prevention of hypoperfusion is of utmost importance in the intraoperative setting, and early detection and treatment are crucial to minimize the risk of organ failure [107]. It is worth noting that some patients may exhibit a positive response to fluid infusion without an immediate need for fluid therapy [108]. In a variety of scenarios, fluid therapy should be guided by macrohemodynamic variables such as stroke volume and dynamic arterial elastance as well as markers of hypoperfusion such as SvcO_2_ and lactic acid [109,110]. Manipulating these variables should result in concurrent improvements in microcirculatory perfusion and tissue oxygenation. This alignment between macrohemodynamics and microcirculation, known as hemodynamic coherence, may not always be present. Excessive fluid administration leading to hypervolemia can compromise the glycocalyx and impair microcirculation, disrupting hemodynamic coherence and impeding oxygen delivery to tissue cells [110].

Crystalloid fluid has an indisputable positive effect on a frequent cause of shock such as hemorrhage, as the restored intravascular volume normalizes the body’s physiology to the pre-bleeding situation. The background is more complex during anesthesia where crystalloid fluid is indicated even if no bleeding occurs. A short explanation for this indication is that general anesthesia reduces the driving pressure for circulation, which is called the mean circulatory filling pressure (MCFP), by some 25% [111]. Both venous return and cardiac output are determined by the difference between MCFP and the pressure in the right atrium (i.e., the central venous pressure). When these flows decrease, the arterial pressure drops and hypoperfusion develops. A more simple way to explain the same chain of events is that the “unstressed” blood volume (which does not contribute to the venous pressure) increases at the expense of the “stressed” blood volume (which creates venous pressure). This adaptation is counteracted by PV expansion which, if necessary, is combined with a vasopressor [112].

Replacement fluids are administered to address fluid deficits that cannot be adequately replenished through oral intake. These deficits can arise from various sources, including drains, stomata, fistulas, hyperthermia, open wounds, and conditions such as polyuria (salt-wasting nephropathy, cerebral salt wasting, osmotic diuresis, or diabetes insipidus). However, data on replacement fluids remain limited. Several recent guidelines recommend matching the composition and the quantity of fluids and electrolytes as closely as possible to the lost fluids. Typically, replacement fluids are isotonic balanced solutions. In cases where there is a significant loss of fluids via the gastrointestinal tract, solutions with a high chloride content, such as saline solution (NaCl 0.9%), may be useful as replacement fluids [113].

Nutritional fluid therapy represents a crucial indication for the administration of intravenous fluids as it plays a significant role in meeting the patient’s nutritional requirements. However, the administration of intravenous fluids for nutritional purposes can lead to fluid overload [114].

Maintenance fluid therapy primarily meets the patient’s baseline needs. During the intraoperative period, maintenance fluid therapy compensates for traditionally overestimated insensible and other losses, including diuresis. Postoperatively, maintenance fluid therapy provides free water and meets the patient’s daily electrolyte and glucose needs [105].

## 6. Phases of Fluid Therapy in the Major Surgery and Intensive Care Settings: Evolution of the Patient’s Volemic Status during the Perioperative Period

**Figure 6:**
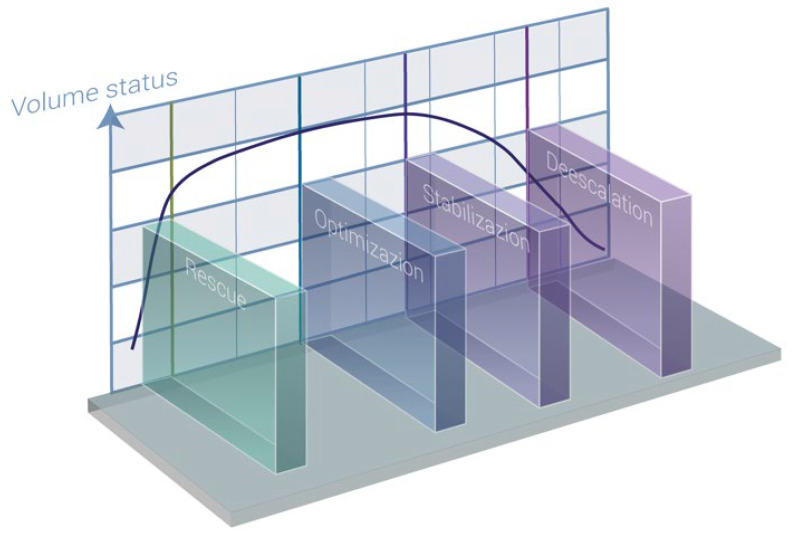
Patient’s volume status at different phases (modified from Hoste et al.) [115].

A fluid therapy protocol consists of two main components: the selection of an ideal fluid and the determination of the appropriate volume for infusion. The administration of fluids often deviates from a linear course due to the dynamic nature of surgical patients. Therefore, the prescription strategy should be adaptable, aiming to detect clinical changes and perioperative complications as early as possible [85]. Two distinct fluid therapy protocols have been defined. The first is the protocol established at the International Fluid Academy Day (IFAD), known as R.O.S.E. (resuscitation, optimization, stabilization, evacuation) [103]. The second is the protocol proposed by the Acute Dialysis Quality Initiative (ADQI), referred to as S.O.S.D. (salvage, optimization, stabilization, de-escalation) [115]. See Figure 6.

The initial phase of any fluid therapy protocol is the rescue phase, which occurs when the patient is in a state of shock. During this phase, the primary objective is to restore the balance between oxygen supply and demand as quickly as possible [116]. Patients in this stage often experience active or recent hemorrhage or have undergone emergency surgery, possibly due to sepsis.

The goal of the optimization phase is to maintain normovolemia, particularly during the intraoperative period. Although intraoperative hypovolemia may not be initially associated with hypoperfusion, studies have linked it to poorer outcomes in surgical patients [117]. During this stage, we typically follow a goal-directed hemodynamic therapy algorithm [118]. The successful implementation of such a strategy necessitates the use of advanced hemodynamic monitoring. Traditional parameters such as heart rate, blood pressure, and diuresis are frequently affected by factors unrelated to the patient’s circulatory status, rendering them unreliable for accurately assessing the patient’s hemodynamic status and reflecting changes in their condition [119].

The stabilization phase differs from the previous two stages by the absence of shock or an imminent threat of shock. At this point, the focus shifts toward providing organ support, and the patient enters a stable steady state. During this phase, fluid therapy is primarily required for ongoing maintenance to compensate for normal fluid losses, such as renal, gastrointestinal, and insensible losses [6]. Replacement fluids may be necessary if the patient is experiencing ongoing losses due to unresolved pathological conditions. It is crucial to note that a persistently positive daily fluid balance over time is strongly associated with a higher mortality rate in septic patients. Therefore, clinicians should be aware of hidden obligatory fluid intake as it can contribute more than one liter of fluid per day [120].

In the maintenance phase, fluids should only be administered to fulfill daily fluid requirements, considering other sources of fluid and electrolytes. If a patient already receives adequate water, glucose, and electrolytes through alternative means such as enteral or parenteral nutrition, medication solutions, and the like, specific intravenous maintenance fluids should be discontinued [121]. It is essential to tailor fluid prescriptions to account for these alternative sources and prevent unnecessary fluid overload [122].

The term “de-resuscitation” was initially proposed in 2012 and officially coined in 2014. It specifically refers to late goal-directed fluid removal and late conservative fluid management [6]. Late goal-directed fluid removal involves an active and assertive approach to removing fluids from the body using diuretics and renal replacement therapy to achieve a negative fluid balance. This approach entails discontinuing invasive treatments and transitioning toward a state of fluid deficit. Late conservative fluid management, on the other hand, involves a more moderate strategy of fluid administration after initial treatment to prevent or reverse fluid overload. Recent studies have highlighted the significance of achieving two consecutive days of negative fluid balance during the first week of intensive care unit (ICU) stay as a robust and independent predictor of survival [123].

Assessing preload responsiveness can still be valuable in determining the initiation and cessation of fluid removal [124]. If no signs of preload responsiveness are detected, it is reasonable to assume that fluid removal will not decrease the cardiac output [70]. Conversely, positive indicators of the preload response may indicate the limits of fluid removal. However, it is crucial to exercise caution during this phase to avoid overly aggressive fluid withdrawal, which can lead to hypovolemia. Hypovolemia carries the risk of hemodynamic deterioration and inadequate tissue perfusion, compromising patient outcomes. Striking the right balance between achieving a negative fluid balance and maintaining adequate fluid volume is essential for optimal management during de-resuscitation [123].

## 7. Clinical Applications of Crystalloids within Enhanced Recovery Pathways

Since the late 1990s, there has been a resurgence in the recognition and exploration of surgical stress, driven by increasing concerns regarding its impact on patient recovery and surgical outcomes. This renewed focus has led to a better understanding of the physiological response to stress and its underlying mechanisms. As a result, a comprehensive set of measures has been devised and compiled aimed at adopting a less metabolically demanding approach during the perioperative period [125].

One prominent manifestation of this approach is the implementation and widespread adoption of Enhanced Recovery After Surgery (ERAS) protocols, which place significant emphasis on perioperative fluid therapy. These protocols have demonstrated remarkable efficacy in reducing surgical morbidity and mortality and hospital stays and overall healthcare costs [2].

### 7.1. Crystalloids as Resuscitation Fluids during the Perioperative Period

In the past three decades, numerous studies have investigated the use of balanced crystalloids compared to 0.9% saline for resuscitation fluids. These studies encompassed controlled trials involving healthy volunteers, observational studies, and clinical trials conducted in surgical settings and with adult critical patients.

The SPLIT trial (0.9% Saline versus Plasma-Lyte 148 for ICU Fluid Therapy) [126] involved 2278 patients undergoing cardiac surgery or during the immediate postoperative period. The primary endpoint of the study was the risk of death. Patients receiving Plasma-Lyte 148 were compared with those who received 0.9% saline before admission to the post-anesthesia care unit. The relative risk of in-hospital mortality for patients receiving balanced crystalloids compared with those receiving 0.9% saline was 0.87 (95% CI, 0.64–1.18). The SALT (Isotonic Solution Administration Logistical Testing) trial [127] included 974 adults admitted from the emergency department, with sepsis being the most common diagnosis. Most patients had received 0.9% saline before admission to the ICU and subsequently received isotonic crystalloids. The odds ratio for 30-day in-hospital mortality in patients receiving balanced crystalloids compared with 0.9% saline was 0.91 (95% CI, 0.64–1.30). The incidence of death, dialysis, or persistent renal dysfunction was lower with balanced crystalloids but higher among patients receiving increased volumes of isotonic crystalloids.

Building upon these studies, two large-scale clinical trials have recently been completed involving nearly 30,000 adults. The SMART (Isotonic Solutions and Major Adverse Renal Events Trial) trial [128] and the SALT-ED (Saline Against Lactated Ringer’s or Plasma-Lyte in the Emergency Department) trial [129] were randomized crossover trials comparing the administration of balanced crystalloids (Ringer Lactate or Plasma-Lyte) with 0.9% saline in critical care and emergency department patients.

The SMART trial [128] recruited 15,802 adult patients from critical care units, with a significant proportion admitted from the emergency department or surgery ward. The primary endpoint was the incidence of severe renal dysfunction, death, dialysis, or persistent renal dysfunction after 30 days. Patients receiving balanced crystalloids had a lower incidence of severe renal dysfunction compared with those receiving 0.9% saline (14.3% vs. 15.4%, *p* = 0.04). In patients with sepsis or septic shock, the 30-day in-hospital mortality was lower in the balanced crystalloid group compared with the 0.9% saline group (25.2% vs. 29.4%, *p* = 0.02). Using balanced crystalloids in patients at higher risk of severe renal damage or death resulted in absolute risk reductions of 3.7% and 4.2%, respectively.

The SALT-ED trial [129] enrolled 13,347 patients who received intravenous crystalloids in the emergency department and were subsequently hospitalized in a non-ICU facility. The primary endpoint was the length of the hospital stay, and the secondary endpoint included major adverse kidney events. Patients receiving balanced crystalloids had similar results in terms of the length of the hospital stay but showed better outcomes regarding major adverse kidney events.

Although the SMART and SALT-ED trials have limitations, such as being single-center studies with a low total volume of fluids infused, they provide valuable insights. Additionally, the SOLAR trial [130] (Saline or Lactated Ringer’s Trial), a cohort study in elective colorectal and orthopedic surgery patients, found no statistically significant differences between the Ringer lactate and 0.9% saline groups regarding postoperative complications and in-hospital mortality.

More recently, the PLUS (Plasmalyte 148 versus Saline) trial [131] and the BaSICS (Balanced Solutions in Intensive Care Study) trial [132] were conducted. The PLUS trial included 5037 patients requiring fluid resuscitation in the ICU and found no statistically significant differences in the onset of renal failure or 90-day mortality between balanced crystalloids and 0.9% saline. The BaSICS trial recruited over 11,000 patients from Brazilian ICUs and found no differences in renal or in-hospital mortality between patients receiving 0.9% saline and those receiving balanced solutions.

An updated meta-analysis [133] incorporating the PLUS and BaSICS trials and 11 other high-quality clinical trials indicated that the use of balanced crystalloids resulted in a relative reduction in mortality ranging from 9% to 1%. The analysis also demonstrated a similar reduction in the risk of renal dysfunction.

In conclusion, the evidence from these studies supports the use of balanced crystalloids as resuscitation fluids during the perioperative period. They have shown potential benefits, including reduced risks of severe renal dysfunction, in-hospital mortality, and major adverse kidney events when compared with 0.9% saline. However, further research is needed to address limitations and provide more robust evidence in specific patient populations and clinical settings.

### 7.2. Crystalloids as Maintenance Fluids during the Preoperative Period

Prolonged preoperative fasting is associated with various harmful effects on surgical patients, including anxiety, dehydration, nausea, vomiting, and impaired insulin resistance during the postoperative phase [134]. Intensified recovery strategies now recommend shorter fasting periods of 6 h for solids and 2 h for fluids [135]. Following these protocols has not been shown to affect the patients’ fluid status or increase their fluid responsiveness as measured via passive leg elevation just before surgery [136].

The preoperative administration of a carbohydrate-rich drink (200–300 mL), primarily consisting of maltodextrins, the night before surgery and up to 2 h preoperatively does not increase gastric volume or pose a risk of aspiration and has been found to enhance postoperative recovery [137]. The routine use of systematic mechanical bowel preparation (MBP) in the pre-operative setting should be avoided [138]. Current evidence supporting its use is limited to specific cases of rectal surgery where a protective stoma is anticipated [139]. With a significant reduction in the number of patients requiring MBP and a shortened preoperative fasting period, prescribing crystalloids as maintenance fluid therapy before elective surgery is physiologically unnecessary [134]. However, a maintenance intravenous infusion of 5% glucose with electrolytes might be considered to improve well-being in fasting patients who are operated on in the late afternoon.

### 7.3. Fluid Therapy Strategies during Surgery

There are three main strategies for the administration of fluid during surgery.

- Fluid balance approach: The anesthetist summarizes measured and perceived losses of fluid during the surgery and replaces the volumes according to the known plasma volume (PV) expansion effects of crystalloid and colloid infusion fluids. This individualized method is the most common worldwide and is appropriate for surgeries of short duration (we suggest <1 h) [140].
- Outcome-based approach: Here, researchers have compared the incidence of postoperative complications in groups of patients randomized to receive one of two pre-determined fluid regimens, such as liberal/restrictive or crystalloid/colloid. A fluid strategy according to this approach is appropriate for intermediate-length surgery (1–3 h) without major hemorrhage.
  Over the past two decades, numerous studies have compared various intraoperative infusion protocols, using multiple definitions of liberal and restrictive regimens. Typically, in major abdominal surgery, a regimen that administers an intraoperative infusion exceeding 7 mL/kg/h is categorized as liberal, while an approach characterized by an infusion rate not surpassing 5 mL/kg/h is considered a restrictive strategy [141,142].
- Goal-directed fluid therapy should be applied for major surgery with an expected larger hemorrhage, such as the Whipple operation or abdominal cancer surgery. The fluid administration is then guided according to invasive hemodynamic measurements whereby the stroke volume is maintained at the flat portion of the Frank–Starling curve. The interested reader is referred to special literature on this topic [143]. Historically, colloid fluids with or without augmentation with vasopressors have been used for this purpose. However, crystalloid fluid is probably most commonly used in the clinical setting today.
  Adhesion to the perioperative protocols for goal-directed fluid therapy can result in volume overload if applied in intensive care. Here, fluid boluses should be provided when signs of poor organ perfusion (liver, kidney, etc.) or hemodynamic instability appear.

### 7.4. Crystalloids as Maintenance Fluids during Intraoperative Period

The need for PV expansion during surgery is mainly due to the previously mentioned reduction in the MCFP. In addition, the excreted urine, bled volume, and insensible fluid losses need to be replaced. The goal is to maintain constancy in the internal environment despite the complex fluid balance situation that is at hand. Traditionally, insensible losses during surgery have been overestimated [75]. This, coupled with the assumption (yet unproven) of a non-functional extracellular space known as the third space [144] and the supposed hypovolemia resulting from preoperative fasting [136], has led to misguided liberal fluid therapy regimens, which have been associated with poorer postoperative outcomes [75]. In theory, balanced crystalloids are the most appropriate choice for intraoperative maintenance fluid therapy due to their ability to distribute within the extracellular space. The term “crystalloid fluid” commonly denotes electrolyte-containing fluid, but solutions based on glucose are also crystalloids. They may be indicated especially in the postoperative period, but only on special indications during ongoing surgery due to the risk of hyperglycemia, which promotes postoperative infection and osmotic diuresis [145,146]. The recommended crystalloid dose is 3 mL/kg/h for laparoscopic surgery and 5 to 7 mL/kg/h for laparotomy and abdominal surgery [85]. Rates of 2 mL/kg/h are associated with an increased incidence of postoperative nausea [147].

### 7.5. Fluid Creep

Fluid creep refers to the amount of fluid (and sodium) administered to dilute intravenously administered drugs, infuse prediluted medications, facilitate catheter flushing, or aid in the advancement of medication through a catheter. A retrospective study conducted in a Belgian ICU involving nearly 15,000 patients concluded that fluid creep accounted for one-third of a critically ill patient’s fluid intake during their ICU stay [148]. However, fluid creep not only contributes to fluid intake but also to sodium intake. Certain prediluted drugs, such as ciprofloxacin, contain dosage forms that include 2.82 g of sodium per daily dose [149], surpassing the maximum recommended daily sodium intake of 2.3 g by the World Health Organization (WHO) or 1.5 g per day in the presence of comorbidities [150]. A 5% glucose solution may serve as a safe alternative for diluting most medications, limiting the sodium content from the saline solution and promoting glycemic control in critically ill patients. Among commonly used drugs, only amoxicillin-clavulanic acid, phenytoin, somatostatin, and acyclovir require dilution in 0.9% saline [114].

### 7.6. Postoperative Maintenance Fluid Therapy

Oral intake should be encouraged as soon as possible, and the administration of intravenous fluids should be limited [125]. However, in certain situations related to the surgery, the patient, or the patient’s inability to adhere to enhanced recovery programs, maintenance fluid therapy may be necessary [151]. The goal of postoperative maintenance fluid therapy is to provide adequate free water and electrolytes to maintain homeostasis in the internal environment and sufficient glucose to prevent a shift toward anaerobic cellular metabolism (ketosis) when oral intake alone is insufficient to meet daily requirements [122]. Daily requirements typically include 25–30 mL/kg of water, 0.5–1 mmol/kg of potassium, 1 mmol/kg of chloride, 1 mmol/kg of sodium, and 50–100 g/day of glucose [121].

A cross-sectional observational study on fluid therapy by Uña et al. revealed that fluid therapy regimens prescribed in clinical practice often fail to meet patients’ daily requirements, frequently providing excessive amounts of sodium and chloride and insufficient amounts of potassium, calcium, and magnesium [152]. Such imbalances in nutrient supply have been associated with poorer postoperative outcomes [71]. Maintenance fluid therapy contributes more fluids, sodium, and chloride to critically ill patients during their ICU stay compared with fluid resuscitation therapy [148]. In pediatric literature, it is emphasized that isotonic maintenance fluids protect children from the morbidity associated with hyponatremia [153]. This population is particularly vulnerable to the development of neurological symptoms due to electrolyte imbalances and to hypoglycemia [113]. However, recommending isotonic maintenance solutions for all hospitalized patients to prevent hyponatremia is likely unnecessary because only a minority of patients are at risk of developing the condition, particularly among adults who typically obtain sodium from multiple sources and have their sodium levels measured frequently during their hospital stay [114]. The use of hypotonic maintenance fluids may be considered a safe alternative, as concluded by two recent studies: one conducted on healthy volunteers [154] and another on post-operative thoracic surgery patients [155]. The results demonstrate that through restricting sodium and chloride intake in patients, a more balanced water balance is achieved, leading to reduced kidney damage as measured via plasmatic neutrophil gelatinase-associated lipocalin (NGALp), in comparison to a group receiving isotonic maintenance fluids. Current evidence does not associate the use of hypotonic maintenance fluids with the occurrence of symptomatic or severe episodes of hyponatremia. However, caution should be exercised in prescribing these fluids to patients admitted with hyponatremia to our unit and to pediatric patients, while they should be completely avoided in patients with cerebral damage [156].

## 8. Conclusions

Perioperative fluid therapy plays a crucial role in determining postoperative outcomes. Crystalloids are the preferred choice of fluids during the perioperative period. An in-depth understanding of these agents and the biochemical and physiological factors governing their in vivo performance is essential for their safe prescription to surgical patients, allowing for the preservation of the dynamics, composition, and homeostasis of the internal environment.

## Figures and Tables

**Table 1:** Composition of plasma and the most common crystalloid solutions.

	NaCl 0.9%	Lactated	Plasmalyte^®^	Isofundin^®^	Ionolyte^®^	5% Glucose	3.3% Glucose	Benelyte^®^	Maintelyte^®^	Plasma
		Ringer’s					NaCl 0.9%			
Na^+^ (mEq/L)	154	131	140	145	137	-	154	140	40	140
K^+^ (mEq/L)	-	5.4	5	4	4	-	-	4	20	4
Ca^2+^ (mEq/L)	-	2	0	2.5	0	-	-	2	-	5
Mg^2+^ (mEq/L)	-	-	1.5	1	1.5	-	-	1.5	3	2.5
Cl^−^ (mEq/L)	154	109	98	127	110	-	154	118	40	98
Bicarbonate (mEq/L)	-	-	-	-	-	-	-	0	23	24
Lactate (mEq/L)	-	28	-	-	-	-	-	0	-	-
Acetate (mEq/L)	-	-	27	24	34	-	-	30	23	-
Citrate (mEq/L)	-	-	-	-	-	-	-	0	-	-
Malate (mEq/L)	-	-	-	5	-	-	-	0	-	-
Gluconate (mEq/L)	-	-	23	-	-	-	-	0	-	-
Glucose (g/L)	-	-	-	-	-	50	33	10	50	-
SID in vivo (mEq/L)	0	28	50	25.5	34	0	0	-	23	42
Osmolarity	308	277	295	309	286.5	278	585	351	402	285–295
(mOsm/L)										

## Data Availability

Not applicable.
